# *Cymbopogon citratus* Allelochemical Volatiles as Potential Biopesticides against the Pinewood Nematode

**DOI:** 10.3390/plants13162233

**Published:** 2024-08-12

**Authors:** Jorge M. S. Faria, Pedro Barbosa

**Affiliations:** 1 INIAV, I.P., National Institute for Agrarian and Veterinary Research, Quinta do Marquês, 2780-159 Oeiras, Portugal; 2 GREEN-IT Bioresources for Sustainability, Instituto de Tecnologia Química e Biológica, Universidade Nova de Lisboa (ITQB NOVA), Av. da República, 2780-157 Oeiras, Portugal; 3 MED, Mediterranean Institute for Agriculture, Environment and Development & CHANGE—Global Change and Sustainability Institute, Institute for Advanced Studies and Research, Évora University, Pólo da Mitra, Ap. 94, 7006-554 Évora, Portugal; pbarbosa@uevora.pt; 4 Centre for Ecology, Evolution and Environmental Changes (CE3C) & Global Change and Sustainability Institute, Faculdade de Ciências da Universidade de Lisboa, Campo Grande, 1749-016 Lisboa, Portugal

**Keywords:** biopesticides, *Bursaphelenchus xylophilus*, citral, emamectin benzoate, essential oil, geraniol, lemongrass, sustainable pest management

## Abstract

Traditional pesticides are based on toxic compounds that can reduce biodiversity, degrade the environment, and contribute to less healthy living. Plant allelochemicals can provide more environmentally friendly and sustainable alternatives. Essential oils (EOs) are complex mixtures of plant secondary metabolites that show strong biological activities. In the present study, the EOs of *Cymbopogon citratus* were screened for activity against the pinewood nematode (PWN), the causal agent of pine wilt disease. To understand their nematicidal properties, EOs were fractioned into hydrocarbon molecules and oxygen-containing compounds, and their main compounds were acquired and tested separately against the PWN. The EO oxygen-containing molecules fraction was highly active against the PWN (EC_50_ = 0.279 µL/mL), with citral and geraniol showing higher activities (EC_50_ = 0.266 and 0.341 µL/mL, respectively) than emamectin benzoate (EC_50_ = 0.364 µL/mL), a traditional nematicide used against the PWN. These compounds were additionally reported to be less toxic to non-target organisms (fish, invertebrates, and algae) and safer to human health (with higher reported toxicity thresholds) and predicted to exert fewer environmental impacts than traditional nematicides. Resorting to approved natural compounds can quickly leverage the development of sustainable alternatives to traditional nematicides.

## 1. Introduction

Aromatic and medicinal plants have been historically used in pharmacies or perfumeries due to the diversity of their chemical compounds. In fact, important advances in medicine can be traced back to the ancient use of plants. They are important sources of essential oils (EOs) that are, nowadays, used for different purposes in industry to give fragrance and taste [1]. At the same time, many biological activities have been attributed to extracts and EOs of aromatic and medicinal plants [2]. EOs are complex mixtures of volatile phytochemicals generally obtained by the hydrodistillation of plants or plant parts. They are commonly composed of terpenes (namely, mono-, sesqui-, and a few diterpenes) and phenolic compounds (mostly phenylpropanoids), although other classes of compounds can also be present in high proportions. Most of these volatiles are secondary metabolites believed to be responsible for important ecological functions in plants, e.g., mediating the interaction between plants and microorganisms, phytophagous pests, or even other plants [3]. Thus, plants have developed a complex arsenal of allelochemicals that are directly responsible for a sophisticated crosstalk between different organisms. These volatiles are an untapped source of potential novel biopesticides with activity against plant parasitic nematodes [4].

The development of sustainable alternatives to traditional pesticides is gaining momentum due to the rise in the number of synthetic compounds and formulations that are being banned or phased out. These dangerous pesticides are the cause of serious environmental and human health concerns. Lately, the fear of pest resistance to traditional pesticides has stimulated the search for more effective alternatives. The EU has set an objective of 50% pesticide reduction in its territory by 2030 [5]; however, between 2011 and 2022, overall pesticide sales were fairly stable, despite strong political and societal pressures for reductions, with some countries reporting a 40% decrease, e.g., Italy and Portugal, but others reporting an almost 80% increase, e.g., Austria and Latvia [6]. This exemplifies the increasing difficulty in controlling plant pests without effective chemical pest management agents.

EOs are good candidates for natural biopesticides because they have strong biological activities against plant pests [7,8,9] and harmless, long-lasting effects on non-target organisms and are easily biodegraded into non-toxic compounds [10]. The genus *Cymbopogon* is one of the most important essential oil-producing Poaceae. Their EOs are among the most widely used in the fragrance industry, e.g., in soaps or aromatherapy candles or as repellents, mainly in sprays or candles for mosquitoes and houseflies. Their characteristic lemony fragrance is the result of a richness in volatile terpenes [11]. Lemongrass (*Cymbopogon citratus*) EOs are dominated by hydrocarbon monoterpenes, such as myrcene or limonene, and oxygen-containing monoterpenes, e.g., the aldehydes citronellal, neral, and geranial and the alcohol geraniol [12]. In natural conditions, the acyclic α, β-unsaturated monoterpene aldehydes geranial (*trans*-citral, citral A) and neral (*cis*-citral, citral B), two geometric isomers, occur as a mixture commonly named citral, with a stronger and sweeter aroma than that of lemon [13] whose biological activity is believed to be directed toward the degradation of cell membranes [14].

Plant parasitic nematodes are plant pests that are difficult to control, given that they commonly infect the inner tissues of plant roots or shoots and spend most of their lives inside the plant or in the soil. The pinewood nematode (PWN), *Bursaphelenchus xylophilus*, is a harmful plant-parasitic nematode that infects the shoots of pine trees, leading to desiccation of the canopy and, eventually, death of the tree (pine wilt disease, PWD). While in its native range, the PWN poses little danger to endemic pine species, its accidental introduction to the susceptible Asian pine forests, and later to Europe, led to massive economic and ecological losses [15,16]. In Japan, losses in timber are reported to average 1 million m^3^/year; in China, over one million trees were killed/year from 1995 to 2006; and in Korea, over one million trees were affected by PWD between 2005 and 2006. In Europe, losses were estimated to be EUR 22 billion over a 22-year period since introduction, with losses in Portugal and Spain being greater than 80% in standing volume [17].

Pest control is commonly performed by restricting wood transport and the application of insecticides and nematicides; however, due to the risk they pose to the environment and human health, the fear of inducing resistance, and a generalized pressure to forego traditional pesticides, sustainable alternatives have already begun to be researched [4,18]. Against PPNs, EOs and EO-derived biopesticides offer many advantages. Besides being environmentally friendlier, they show a wide range of activities, e.g., bactericidal and fungicidal, which can reduce infections by opportunistic microorganisms. They can interact with multiple targets due to their complex composition, reducing the risk of the development of resistance, and their volatile compounds can show synergistic and antagonistic interactions, which allow for the optimization of their nematicidal efficacy [3,4,19]. The known mechanisms of action of EO volatiles against PPNs include disrupting their nervous systems, changing plasma membrane permeability, influencing the gelatinous matrix of PPN eggs, and altering intracellular redox conditions [19]. *Cymbopogon* and *Chrysopogon* are the only Poaceae whose EOs have been tested against the PWN. Those extracted from *Cymbopogon citratus* and *C. martini* show high activities, which makes them strong candidates for the formulation of sustainable bionematicides [4,8,20].

The present study aimed to understand the nematicidal activity of *Cymbopogon citratus* EO against the PWN by analyzing its volatile composition, fractioning its components, and screening its major compounds. Also, its suitability for the formulation of bionematicides was gauged by assessing the potential impacts on the environment and human health.

## 2. Results

### 2.1. Lemongrass Essential Oil Composition

The essential oils obtained from fresh shoots (FS) or dried shoots (DS) of *C. citratus* showed an overall similar qualitative chemical composition. However, a major difference was observed in the proportions of the alcohol geraniol between the samples. The EOs obtained from FS showed a lower proportion of geraniol (0.5 ± 0.1%) than the EOs of DS1 and DS2, where geraniol was one of the components with the highest relative amounts (13.8 ± 1.5 and 17.7 ± 0.3%, respectively). This increase may have been related to the drying process. Large differences were also observed between the commercialized samples (DS1 and DS2). The EO obtained from the dried shoots provided by the retailer in the northern mainland (DS1) showed a higher proportion of monoterpene hydrocarbons than the dried shoots acquired from the retailer in the central mainland (DS2) (Table 1). Concurrently, relative amounts of oxygen-containing monoterpenes were higher for DS2 than for DS1.

The ratio of β-myrcene/neral/geranial (the main compounds common across all samples) for the EO of FS (1.0:1.2:1.7) was more similar to that of DS2 (1.0:1.1:1.7) than DS1 (1.9:1.0:1.2). The hydrocarbon β-myrcene dominated the EO of DS1, while the EOs of FS and DS2 were dominated by the aldehydes neral and geranial (stereoisomers whose mixture is known as citral).

### 2.2. Nematicidal Activity of Lemongrass EOs

The EOs were tested against suspensions of mixed life stages of the PWN. The highest activity was obtained for DS2 EO (0.275 ± 0.002 µL/mL), followed by FS EO (0.429 ± 0.005 µL/mL), with the lowest activity obtained for DS1 EO (0.777 ± 0.003 µL/mL) (Table 2). Compared to the nematicidal activity of emamectin benzoate, the active principle of a commercial nematicide used against the PWN (0.364 ± 0.003 µL/mL), only the EO of DS2 reached a higher activity.

**Figure 1:**
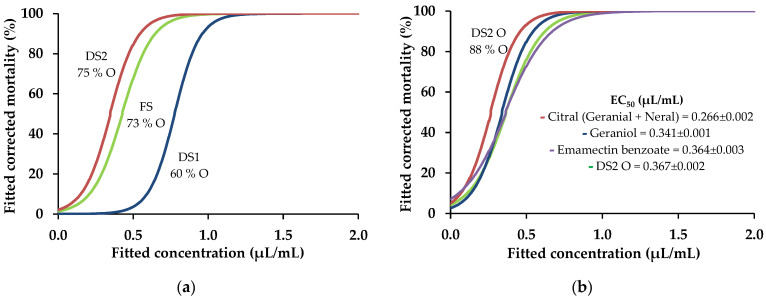
Sigmoidal curves fitted to the nematicidal activity of essential oils from FS, DS1, and DS2 against the pinewood nematode, with respective relative amounts of total oxygen (O)-containing compounds (**a**). For the oxygen-containing compounds fraction (DS2 O), nematicidal activity was compared to that of its main compounds, citral and geraniol, and the commercial nematicide emamectin benzoate (**b**). Half maximal effective concentration (EC_50_) values are shown for comparison purposes.

Nematicidal activity seemed to increase with EO richness in oxygen-containing compounds (Figure 1a). The EO of DS2 was further analyzed to identify the origin of its nematicidal properties. Through fractionation, DS2 EO was partitioned into a hydrocarbon-enriched fraction (DS2 H) and a fraction enriched with oxygen-containing compounds (DS2 O) and tested separately against the PWN. The volatile profile of DS2 H showed a higher proportion of hydrocarbon terpenes (74%), but some oxygen-containing compounds could still be found (11%). Similarly, DS2 O was mainly composed of oxygen-containing compounds (89%), but some hydrocarbon molecules were still detected (1%), suggesting that the fractionation process was successful but not fully effective. Against the PWN, the DS2 H fraction showed only moderate mortality (50.3 ± 1.6%) at the highest concentration tested, 2 µL/mL (Table 2). On the other hand, the DS2 O fraction was highly active and reached an EC_50_ value (0.279 ± 0.002 µL/mL) similar to that of the original EO (DS2 = 0.275 ± 0.002 µL/mL), suggesting that the compounds in this fraction may be mainly responsible for the nematicidal activity of the original EO.

**Figure 2:**
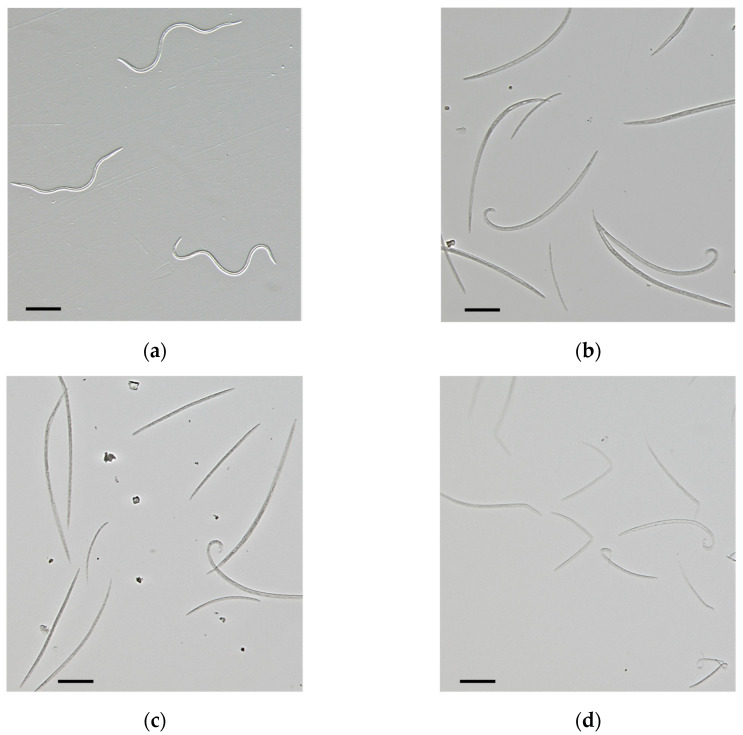
Microscopic detail of pinewood nematodes in control wells (**a**) and in wells with 1 µL/mL of geraniol (**b**), citral (**c**), or emamectin benzoate (**d**). PWNs killed by geraniol or citral showed a typical extended body shape (**b**,**c**), while those affected by emamectin benzoate showed a twisted body shape (**d**). Bar = 20 µm.

To understand the contribution of the dominant compounds in DS2 O to its overall nematicidal activity, pure commercial standards of citral (ca. 80% of DS2 O fraction), the mixture of the stereoisomers geranial (*trans*-citral) and neral (*cis*-citral) commonly found in natural conditions, and geraniol (ca. 5% of DS2 O fraction), were acquired and separately tested against the PWN (Figure 2).

Both compounds showed a high activity, which indicated that they could be responsible for this fraction’s nematicidal activity (Figure 1b and Figure 2c,d). The experimental values of DS2 O fraction activity against the PWN were compared to the theoretical addition of the singular activities of citral and geraniol at their respective relative amounts in the fraction (Table 3). At each concentration, the combined activities of citral and geraniol were greater than the experimental values obtained, which suggested that compounds with minor proportions in the fraction may interact antagonistically with these compounds on nematicidal activity against the PWN.

### 2.3. Potential Impacts on the Environment and Human Health

To gauge the potential environmental benefits of using *C. citratus* EOs, data on ecotoxicological and toxicological parameters of their main volatiles were retrieved from reputed online repositories, and specialized software was used to understand the main environmental compartments influenced by their release in comparison to the commercial nematicide emamectin benzoate.

Due to their physicochemical characteristics, citral and geranial were predicted to mainly disperse into air (43 and 16%, respectively) and water (48 and 70%, respectively) environmental compartments (Table 4). On the other hand, emamectin benzoate was predicted to have a higher affinity to the soil environmental compartment (98%).

Compound half-life was predicted to be shorter in air than in water, soil, or sediment environmental compartments (Table 4). Citral and geraniol seemed to be potentially retained for a longer period in the water compartment, while emamectin benzoate seemed to be retained in the soil environmental compartment. Emamectin benzoate half-life was predicted to be longer in all compartments, except for the air environmental compartment. Compound persistence in the environment was predicted to be over 3× higher for emamectin benzoate than for citral or geraniol (Table 4).

The toxicity of citral and geraniol to aquatic organisms was reviewed, given that their predicted preferential site of accumulation was the water environmental compartment. The acute toxicity thresholds reported for citral or geraniol were much higher than the ones reported for emamectin benzoate. For fish, LC_50/96h_ values were 34- and 110-fold higher; for invertebrates, EC_50/48h_ values were 6800- and 16,100-fold higher; and for algae, EC_50/48h_ values were over 14,800- and 6800-fold higher, respectively (Table 5).

Lastly, the benefits of citral or geraniol were assessed by reviewing the acute toxicity thresholds for oral and dermal routes of exposure for mammals in comparison to the nematicide emamectin benzoate. The values of LD_50_ reported for citral or geraniol through the oral route of exposure were much higher than that reported for emamectin benzoate (ca. 60- or 44-fold higher, respectively) (Table 6). For the dermal route of exposure, the values of LD_50_ reported for citral or geraniol were closer to that reported for emamectin benzoate, but still 5- and 11-fold higher, respectively. These parameters suggest greater safety to human health in using these monoterpenes than the commercial nematicide.

## 3. Discussion

The development of novel plant protection products that can be sustainably produced, have fewer environmental impacts, and are safer for human health is the challenge currently being faced. EOs provide good candidates since they can be obtained by simple distillation processes from plant material and have strong biological activities as they are composed of several allelochemicals with diverse chemical properties. Against the PWN, the EOs of *C. citratus* showed a high nematicidal activity that appeared to be dependent on chemical composition. The EOs assayed in the present study showed variation in the proportions of the volatiles but not in their chemical profiles. The EO with the lowest activity was richer in monoterpene hydrocarbons, mainly *β*-myrcene, while those with the highest activities were richer in oxygen-containing volatiles, being dominated by citral (neral and geranial) and geraniol, irrespective of having been obtained from fresh or dried material. For obtaining EOs, the use of dried material is commonly favored since it can yield higher amounts of EO per kg of material, which can reduce production costs. In previous studies on the activity of *Cymbopogon* EOs against the PWN, complete mortality was also observed for *C. citratus* at 2 and 0.5 mg/mL. At a lower concentration, females and juveniles showed ca. 50% mortality, but males were more resistant, showing ca. 30% mortality at 0.25 mg/mL [7]. For *C. nardus* EO, at 2 mg/mL, no mortality was observed [7]. Although these authors did not specify the volatile profiles of the EOs, in similar studies on the activity of EOs against the root-knot nematode *Meloidogyne incognita*, *C. nardus* EOs showed substantial chemical variability, with some being rich in citral, myrcene, and geraniol, while others showed dominance of citronellal, geraniol, and citronellol, which impacted their nematicidal activities [27,28]. For *C. citratus* EOs tested against the PWN, EC_50,24h_ values varied between 0.287 and 0.456 µL/mL [8,20,29].

Thus, the choice of chemotype is also an important factor that should be consistent in order to maintain an EO profile with high amounts of oxygen-containing monoterpenes, which seem to be responsible for higher nematicidal activity against the PWN. In the present study, EO fractionation allowed us to determine the contribution of the hydrocarbon molecules in comparison to the oxygen-containing molecules. The first showed some activity (ca. 50% mortality at the highest concentration), undoubtedly also caused by the presence of residual oxygen-containing molecules (11%) as a result of an incomplete fractionation process. The fraction containing the highest amount of oxygen-containing molecules (89%) was as successful as the original EO (DS2), showing only slightly higher EC_50_ and lower EC_20_ and EC_100_ values, which suggests that PWN response to increasing EO concentrations can be faster in the absence of hydrocarbon molecules. In a previous study, the activity of *C. citratus* EO was also seen to be equally strong against the PWN, as was its oxygen-containing molecules fraction, despite synergistic and antagonistic interactions being identified between the fractions of EOs of other active plants [20].

The dominant volatiles (citral and geraniol) in the oxygen-containing molecules fraction were tested separately to understand their contribution to overall nematicidal strength. Citral showed strong activity against the PWN that surpassed that of geraniol. Indeed, in similar studies, geraniol showed an EC_50_ value that was higher (0.470 mg/mL) than that of citral (0.187, 0.139, and 0.110 mg/mL for PWN males, females, and juveniles, respectively) [30,31]. In a different study, the stereoisomers that form citral were tested separately, and geranial (0.120 mg/mL) showed stronger activity than neral (0.525 mg/mL) [30,32], which further suggests that the isomer composition of citral can also influence its nematicidal activity. In these molecules, the aldehyde appears to exert a stronger nematicidal influence than the alcohol since nerol also showed higher EC_50_ values, namely, 0.865, 0.926, and 0.979 mg/mL for PWN males, females, and juveniles, respectively, than neral [31].

The potential impacts of these phytochemical volatiles on the environment and human health were gauged by resorting to toxicological and ecotoxicological online databases and specialized software. In comparison to emamectin benzoate, citral or geraniol, as main compounds of *C. citratus* EO, showed affinity to air and water environmental compartments, instead of the soil environmental compartment. They should be less persistent than this pesticide in any of these compartments. Indeed, citral and geraniol are reported to be readily biodegradable in water, with degradation of ca. 70 and 98% after 28 days, respectively, whereas emamectin benzoate is reported not to be readily biodegradable, exhibiting 0% degradation after 28 days [26]. This is of high importance when the toxicity thresholds reported for aquatic model organisms are taken into consideration. Being less degradable, emamectin benzoate can accumulate in higher amounts and induce higher mortality in natural populations since its acute toxicity thresholds to aquatic organisms are very low in comparison to citral or geraniol. These phytochemicals degrade faster, accumulate less, and are less toxic to aquatic organisms, which makes them a lower potential environmental risk.

Regarding their safety for human health, both citral and geraniol have reportedly higher acute toxicity thresholds through the oral and dermal routes of exposure than emamectin benzoate. Using the globally harmonized system (GHS) of classification and the labelling of hazardous substances, where compounds are assigned to one of five hazard categories according to numeric cut-off criteria [33], citral and geraniol can both be classified as category 5 molecules, while emamectin benzoate is a class 3 toxicant for both oral and dermal routes of exposure. This suggests potentially greater safety in handling these phytochemicals in comparison to emamectin benzoate.

The use of EOs and their volatiles has many advantages for the formulation of biopesticides, such as the multiple biological activities and high effectiveness they exhibit, their low toxicity to non-target organisms, low persistence in the environment, and low production costs. However, their use in commercially manufactured biopesticides is still very low, probably due to (a) drawbacks on the complexity and costly authorization processes to legitimize new botanical pesticides based on plant extracts with no proven history of use in the food industry, cosmetics, or medicine; (b) the low stability in natural conditions, which leads to loss of efficiency; and (c) a generalized lack of quality and security on EO composition as well as a dedicated industry that can provide a sufficient quantity of material at affordable prices [34]. Nevertheless, by providing sound scientific studies with practical results that deal with the environmental risks and impacts on non-target organisms, this field can be leveraged and promote the development of new biopesticides with safer environmental properties.

## 4. Materials and Methods

### 4.1. Essential Oils, Fractions, and Volatiles

Fresh lemongrass aerial parts (FS) were collected in the spring in the vicinity of Campo Grande, Lisbon. Dried aerial parts commonly commercialized in Portugal were acquired from a retailer in northern (Viseu) (DS1) and another in central mainland (Lisbon) (DS2). Essential oils were obtained through hydrodistillation for 3h according to the European Pharmacopoeia [35] and analyzed with gas chromatography coupled mass spectrometry (GC-MS) [29]. To isolate the fraction of the EO enriched with hydrocarbon molecules from the fraction enriched with oxygen-containing compounds, column chromatography was used to fraction 5 mL of EO on a 22 g silica gel (Silica gel 60, Merck 9385) column (380 mm length and 85 mm internal diameter) by elution with 20 mL of distilled *n*-pentane (Riedel-de Haën, Sigma-Aldrich, Steinheim, Germany) per mL of EO to elute hydrocarbon molecules, followed by elution with 20 mL diethyl ether (Panreac Química S.A.U., Barcelona, Spain) per mL of EO to elute oxygenated molecules. Each fraction was first concentrated under reduced pressure on a rotary evaporator (Yamato, Hitec RE-51) and then under nitrogen flux and, finally, stored in the dark at −20 °C until analysis. The volatiles citral (mixture of *cis* and *trans*, purity ≥ 96%) and geraniol (purity ≥ 98.5%) were pure analytical standards acquired from Sigma-Aldrich (St. Louis, MO, USA). The HPLC-grade solvent methanol, used for stock solutions of the EOs and volatiles, was acquired from Fisher Chemicals (Newington, NH, USA). The commercial nematicide emamectin benzoate (Pursue^®^, Syngenta, Lisbon, Portugal) was also tested.

### 4.2. Rearing the Pinewood Nematode

The PWN was isolated from a *Pinus pinaster* field tree displaying strong PWD symptomatology (N 39°43′338″, W 9°01′557″). This isolate was identified as Bx0.13.003 and kept as a reference isolate at the Plant Nematology Lab of the National Institute for Agrarian and Veterinary Research (INIAV, I.P.) at Oeiras, Portugal, with an internal transcribed spacer (ITS) region sequence being deposited in the GenBank database (NCBI) with the accession number MF611984.1. Under laboratory conditions, PWNs were cultured in vitro using a non-sporulating *Botrytis cinerea* (de Bary) Whetzel strain in aseptic conditions. For direct-contact bioassays, larger quantities of PWNs were needed, so axenic cultures of *B. cinerea* were established on steam-sterilized, hydrated, certified, organic, commercial barley grains (*Hordeum vulgare* L.) (ca. 15 g cereal/15 mL ultrapure water in 250 mL Erlenmeyer flasks) for 7 to 10 days at 25 ± 1 °C [20]. Following this, 1 mL of a mixed life-stage PWN suspension (1000 PWNs/mL) was added, and the cultures were kept at 25 ± 1 °C in darkness for 7 to 10 days until the fungal mat was consumed by the PWNs. Nematodes were extracted using the modified Baermann funnel technique [36]. Aqueous solutions of PWNs were used for the direct-contact assays or further inoculations or stored at 11 °C. The assessment of PWN numbers and/or survival rates was performed using an Olympus SZX12 (Tokyo, Japan) stereomicroscope (40×).

### 4.3. In Vitro Direct-Contact Bioassays

The nematicidal activity of the essential oils, their fractions, pure volatile standards, and emamectin benzoate was screened through direct-contact bioassays. Stock solutions were prepared in HPLC-grade methanol at 40 µL/mL. In flat-bottom 96-well microtiter plates (Carl Roth GmbH & Co. KG, Karlsruhe, Germany), 95 µL of an aqueous suspension containing 80 to 100 mixed life-stage PWNs was added to 5 µL of stock solution per well to obtain a final concentration of 2 µL/mL [37]. Lower concentrations were obtained by serial dilutions with a dilution factor of two for final concentrations of 1, 0.5, 0.25, and 0.125 µL/mL. For each microtiter plate, blank wells were performed to assess natural PWN mortality by adding 5 µL of ultrapure water instead of the stock solution and having control wells with 5 µL of methanol to determine the contribution of methanol to PWN mortality. Plates were then sealed with plastic film to prevent EO volatilization and mixed in an orbital shaker (IKA labortechnik, Staufen, Germany) at 800 cycles/min for 1 min. Aluminum foil was used to cover the plates, which were kept at 25 ± 1 °C for 24 h in an orbital shaker at 50 r.p.m. After this time, live and dead PWNs were counted under a stereomicroscope (40×). Mortality was ascertained by the physical prodding of motionless PWNs. Three separate trials were performed for each sample in a total of 10 bioassays.

### 4.4. Prediction of Environmental and Ecotoxicological Parameters

To understand the potential dispersal of the EO compounds to the environmental compartments, the predictive equilibrium criterion model suggested by Mackay et al. [22] was used to compare these compounds to the synthetic nematicide emamectin benzoate. Following this model, 100,000 kg of the compound (simulating an accidental spill into the environment) was hypothetically introduced to a closed system, under steady-state and equilibrium conditions, at a temperature of 25 °C, and its potential environmental fate was predicted for the air, water, soil, and sediment compartments by using the freely available Level I Mackay Fugacity Model beta version 4.31, Trent University, Canada [21]. For each compound, the physicochemical parameters needed to determine the partition ratios were the molecular mass (g/mol), melting point (°C), vapor pressure (Pa), solubility in water (mg/L), air–water partition coefficient or Henry’s Law constant (Pa/mol/m^3^), *n*-octanol/water partition coefficient (log value of Kow), and soil organic carbon/water partition coefficient (Koc), which were retrieved from the PubChem online database [24] and PPDB: the Pesticide Properties Database [25] (Table 7).

For the prediction of compound persistence and half-life, EPISuite™ software (v. 4.11) [23], freely available from the US Environmental Protection Agency (Washington, DC, USA), was used.

### 4.5. Reported Toxicological and Ecotoxicological Acute Toxicity Thresholds

The assessment of potential risks to human health was performed by resorting to data on animal studies where acute toxicity thresholds were experimentally determined, mainly through the oral and dermal toxicity routes. Acute oral toxicity is commonly expressed as the dose of a compound that is administered through the mouth and is lethal to 50% of the tested animals within a specified time (LD_50_). Acute dermal toxicity determines the median lethal dose (LD_50_) causing toxic effects through skin exposure.

For the assessment of environmental risks to aquatic organisms, toxicity thresholds are generally obtained on model organisms for three important trophic levels: primary producers, such as algae or plant species; primary consumers/secondary producers, using invertebrates such as *Daphnia* spp.; and secondary consumers, screening toxicity on vertebrates, namely, species of fish.

For the present work, experimental data were retrieved from reputed databases that host databases for these parameters, namely, the European Chemicals Agency (ECHA) database [26], PPDB: the Pesticide Properties Database [25], and PubChem [24].

### 4.6. Data Treatment and Statistical Analysis

Live and dead PWN counts were used to determine mortality percentages according to Formula (1):

Mortality % = (dead PWNs/total no. of PWNs) × 100(1)

For EOs, fractions, and compounds, mortality percentages were corrected to exclude control mortality through Formula (2):

Corrected mortality % = [(mortality % in treatment − mortality % in control)/(100 − mortality % in control)] × 100(2)

The toxicological strength of an EO or compound was characterized as complete when mortality was 100%, strong when above 80%, moderate when between 80 and 61%, weak when between 60 and 40%, and low or inactive when below 40% [8].

To determine half maximal effective concentration (EC_50_) values, Version 2019 of Origin Graphing & Analysis software (OriginLab, Northampton, MA, USA) was used. A nonlinear regression analysis was performed by plotting corrected mortality values along EOs, fractions, or compound concentration values and fitting a dose–response log-logistic equation:

y = A1 + (A2 − A1)/1 + exp {[log (x) − log (EC_50_)]*p*} (3)

where A1 and A2 are the lower and upper limits of the sigmoidal dose–response curve, respectively; *p* is the slope and EC_50_ is the EO concentration that induces a response halfway between the lower and upper limits. The upper (A1) and lower (A2) limits were set to 0 and 100%, respectively. Determination of the lowest maximal effective concentration (EC_100_) was performed by solving the curve equation to the first y value of 100% mortality, while the fitted mortality values of the compounds were determined by solving the curve equation to the x value at their respective concentrations (ratio in the EO multiplied by EO concentration in the well).

## 5. Conclusions

Essential oils and their main volatiles are sources of new biopesticide formulations that have the advantages of high activities against certain groups of pests and low toxicity to non-target organisms. Against the PWN, the EO of *C. citratus* was very active, depending on its volatile profile. Higher amounts of oxygen-containing molecules are required for increased activity. Its main oxygen-containing compounds were more active than the commercial nematicide emamectin benzoate and showed more favorable environmental and human health-related properties. Its use can be facilitated due to it already being an approved insecticide, but its stability in EO compositions should be assured for a commercial product to be developed.

## Figures and Tables

**Table 1:** Main composition of volatiles (≥1%) in the essential oils extracted from *Cymbopogon citratus* freshly collected shoots (FS) or dry shoots acquired from commercial sources in northern (DS1) and central (DS2) mainland Portugal.

Compounds (≥1%)	FS	DS1	DS2	DS2 H ^1^	DS2 O ^1^
**Hydrocarbons**					
*Monoterpenes*	**25.1**	**38.2**	**19.5**	**74.0**	**1.4**
β-Myrcene	24.7 ± 2.6	38.2 ± 4.4	19.5 ± 1.9	72.9 ± 0.3	1.4 ± 0.1
*cis*-β-Ocimene	0.4 ± 0.0	t	t	1.1 ± 0.0	
*Sesquiterpenes*	**0.1**	**0.5**	**2.2**	**1.7**	
*trans*-α-Bergamotene	0.0 ± 0.0	0.4 ± 0.1	1.0 ± 0.0	0.9 ± 0.1	
β-Caryophyllene	0.1 ± 0.0	0.1 ± 0.0	1.2 ± 0.0	0.8 ± 0.1	
**Oxygen-containing compounds**					
*Monoterpenes*	**73.3**	**59.5**	**73.8**	**10.9**	**88.9**
Geranial	42.5 ± 1.3	22.6 ± 1.2	33.7 ± 0.8	1.6 ± 0.1	45.4 ± 0.5
Geraniol	0.5 ± 0.1	13.8 ± 1.5	17.7 ± 0.3		5.2 ± 0.1
Geranyl acetate	0.1 ± 0.0	0.5 ± 0.1	0.3 ± 0.1	0.3 ± 0.0	1.3 ± 0.0
Linalool	0.7 ± 0.1	2.6 ± 0.5	0.2 ± 0.0		1.3 ± 0.1
Neral	28.6 ± 0.7	19.6 ± 0.8	21.5 ± 1.1	9.0 ± 0.1	35.7 ± 0.1
*trans*-Verbenol	1.0 ± 0.1	0.4 ± 0.0	0.4 ± 0.1		
*Sesquiterpenes*	**0.1**	**0.3**	**1.6**		
10-epi-γ-Eudesmol	0.1 ± 0.1	0.3 ± 0.0	1.6 ± 0.1		

t—traces, below 0.05%. ^1^ Due to its high nematicidal activity against the PWN, the EO from DS2 was fractioned into a hydrocarbon compounds fraction (DS2 H) and oxygen-containing molecules fraction (DS2 O).

**Table 2:** Half maximal effective concentration (EC_50_), EC_20_ (average ± standard error), and EC_100_ (upper and lower 95% confidence limits) obtained by fitting a dose–response curve to data of mortality caused by the essential oil of *Cymbopogon citratus* freshly collected shoots (FS) or dry shoots acquired in northern (DS1) or central (DS2) mainland Portugal, their main compounds citral and geraniol, and the commercial nematicide emamectin benzoate. Curve slope (*p*) and goodness of fit (R^2^) are presented for comparison purposes.

	FS	DS1	DS2	DS2 O ^1^	Citral ^2^	Geraniol	Emamectin Benzoate
EC_50_ (µL/mL)	0.429 ± 0.005	0.777 ± 0.003	0.275 ± 0.002	0.279 ± 0.002	0.266 ± 0.002	0.341 ± 0.001	0.364 ± 0.003
*p*	4.328 ± 0.272	5.144 ± 0.069	4.787 ± 0.113	3.707 ± 0.044	4.822 ± 0.097	4.626 ± 0.022	3.081 ± 0.524
R^2^	0.99	0.99	0.97	0.99	0.97	0.99	0.96
EC_20_ (µL/mL)	0.290 ± 0.003	0.660 ± 0.006	0.222 ± 0.003	0.205 ± 0.001	0.141 ± 0.001	0.210 ± 0.000	0.168 ± 0.001
EC_100_ (µL/mL)	1.307–1.916	1.912–1.968	1.524–1.646	1.419–1.467	1.443–1.550	1.626–1.648	1.612–1.702

^1^ The oxygen-containing molecules fraction obtained from the EO of DS2. ^2^ Citral occurs as a mixture of the stereoisomers geranial (*trans*-citral) and neral (*cis*-citral), more commonly found in natural conditions.

**Table 3:** Comparison of corrected mortality values (%) obtained for the oxygen-containing molecules fraction of *Cymbopogon citratus* essential oil (DS2 O) at 2, 1, 0.5, 0.25, and 0.125 µL/mL, with the fitted corrected mortality values for its main volatiles (citral and geraniol) at their relative amounts in the fraction.

Concentration (µL/mL)	2	1	0.5	0.25	0.125
Experimental mortality (%)					
*C. citratus* (DS2) O ^1^	100.0 ± 0.0	99.5 ± 0.1	75.7 ± 0.7	26.9 ± 0.9	11.2 ± 0.6
Mortality from fitted curves (%)					
Citral (geranial + neral), at 81%	100.0–100.0	99.7–99.8	81.3–83.8	32.3–34.1	13.1–14.7
Geraniol, at 5%	7.3–7.6	4.3–4.5	3.3–3.5	2.9–3.1	2.7–2.9
Sum of compounds activity ^2^	>100	>100	84.5–87.3	35.2–37.1	15.8–17.5

^1^ Obtained through column chromatography of *C. citratus* dry shoots essential oil. ^2^ Addition of the fitted corrected mortality values for citral and geraniol at their relative amounts in the fraction.

**Table 4:** Predicted environmental distribution (PED, %), half-life in each environmental compartment (h), and persistence (h) of the aldehyde citral and alcohol geraniol in comparison to the commercial nematicide emamectin benzoate, determined through the fugacity model proposed by Mackay et al. [21,22] and computed using EPISuite software v. 4.11 [23].

Compartments	Air		Water		Soil		Sediment		
Compounds	Amount (%)	Half-Life (h)	Amount (%)	Half-Life (h)	Amount (%)	Half-Life (h)	Amount (%)	Half-Life (h)	Persistance (h)
Citral	42.9	0.47	48.2	360	8.7	720	0.2	3240	374
Geraniol	16.4	0.26	69.7	360	3.6	720	0.3	3240	424
Emamectin benzoate	0.0	0.14	0.1	4320	97.6	8640	2.2	3890	1390

**Table 5:** Acute toxicity thresholds reported for aquatic model organisms (fish, invertebrates, and algae) treated with the aldehyde citral or alcohol geraniol compared to those reported for the commercial nematicide emamectin benzoate [24,25,26].

Acute Toxicity Thresholds	Fish (LC_50/96h_, mg/L)	Invertebrates ^1^ (EC_50/48h_, mg/L)	Algae (EC_50/72h_, mg/L)
Citral	6.8 ^2^	6.8	103.8 ^3^
Geraniol	22.0 ^4^	16.1	48.0 ^5^
Emamectin benzoate	0.2 ^4^	0.001	0.007 ^5^

^1^ Values reported for the model organism *Daphnia magna*. ^2^ Model organism not reported. ^3^ Reported for *Scenedesmus subspicatus*. ^4^ Reported for *Oncorhynchus mykiss*. ^5^ Reported for *Pseudokirchneriella subcapitata*.

**Table 6:** Reported oral and dermal acute toxicity thresholds (LD_50_, mg/kg) of the aldehyde citral or alcohol geraniol compared to those of the commercial nematicide emamectin benzoate.

Compounds	Toxicity Thresholds (LD_50_ in mg/kg)	
	Oral	Dermal
Citral	4960	2250 ^1^
Geraniol	3600	>5000
Emamectin benzoate	82	439

^1^ Value reported for rabbit model organism, while the remaining were reported for rat.

**Table 7:** Physical and chemical properties of citral, geraniol, and emamectin benzoate molecules required to perform the Level I Mackay Fugacity Model [21] [molecular mass (g/mol), melting point (°C), vapor pressure (mPa), solubility in water (mg/L), air–water partition coefficient or Henry’s Law constant (Pa/m^3^/mol), *n*-octanol/water partition coefficient (logKow), and soil organic carbon/water partition coefficient (Koc)]. Data were retrieved from the PubChem database [24] and PPDB: the Pesticide Properties Database [25].

Physicochemical Parameters	Citral ^1^ (Geranial + Neral)	Geraniol	Emamectin Benzoate
CAS number	5392-40-5	106-24-1	155569-91-8
Molecular mass (g/mol)	152.23	154.25	1008.2
Melting point (°C)	−10.0	−15.0	146.0
Vapor pressure (Pa)	11.999	3.999	5 × 10^−6^
Solubility in water (mg/L)	1340.0	100.0	24.0
Henry’s law Constant (Pa/m^3^/mol)	4.408	1.165	0.0002
LogKOW (unitless)	3.45	3.56	5.00
KOC (unitless)	83	90	3.78 × 10^5^

^1^ Citral occurs as a mixture of the two geometric stereoisomers geranial (*trans*-citral) and neral (*cis*-citral), more commonly found in natural conditions.

## Data Availability

The raw data are available from the corresponding author (Jorge M. S. Faria) upon reasonable request.
